# Table of Infantile Mortality in the City of New York

**Published:** 1866-07

**Authors:** Cyrus Ramsay


### ART. IV.—*Table of Infantile Mortality in the City of New York.* By CYRUS RAMSAY, M. D., LL.B.

*A Table of Still-born in each year, and Deaths of Infants from one day to one year of age, from* 1854 *to* 1866.

| YEARS. | STILL-BORN ...... | 2 days......... | 2 to 5 days ...... | 5 to 15 days...... | 15 to 21 days ... | 20 days to 1 month | 1 to 2 months...... | 2 to 3 months.... | 3 to 4 months... | 4 to 5 months ... | 5 to 6 months ... | 6 to 7 months ... | 7 to 8 months ... | 8 to 9 months ... | 9 to 10 months.... | 10 to 11 months .. | Under 1 year..... |
|---|---|---|---|---|---|---|---|---|---|---|---|---|---|---|---|---|---|
| 1855............. | 1565 | 351 | 921 | 375 | 113 | 449 | 403 | 814 | 820 | 710 | 412 | 200 | 176 | 298 | 460 | 301 | 6771 |
| 1856............. | 1680 | 302 | 852 | 344 | 93 | 387 | 361 | 786 | 799 | 690 | 382 | 189 | 166 | 263 | 468 | 355 | 6437 |
| 1857............. | 1692 | 387 | 912 | 366 | 143 | 409 | 377 | 794 | 812 | 733 | 402 | 215 | 201 | 303 | 482 | 369 | 6905 |
| 1858............. | 1700 | 462 | 954 | 394 | 160 | 4[illegible]0 | 380 | 794 | 814 | 739 | 408 | 218 | 207 | 310 | 486 | 377 | 7109 |
| 1859............. | 1704 | 424 | 904 | 364 | 132 | 423 | 352 | 753 | 769 | 717 | 368 | 177 | 169 | 271 | 438 | 333 | 6599 |
| 1860............. | 1671 | 376 | 854 | 334 | 104 | 396 | 324 | 712 | 724 | 695 | 328 | 156 | 146 | 241 | 398 | 302 | 6087 |
| 1861............. | 1690 | 400 | 862 | 334 | 108 | 406 | 326 | 730 | 721 | 706 | 340 | 160 | 147 | 346 | 40[illegible] | 303 | 6189 |
| 1862............. | 1660 | 376 | 849 | 321 | 88 | 374 | 334 | 670 | 734 | 374 | 318 | 126 | 136 | 200 | 521 | 309 | 5720 |
| 1863 ............. | 1651 | 398 | 873 | 301 | 102 | 399 | 359 | 737 | 746 | 463 | 352 | 177 | 165 | 241 | 478 | 327 | 6118 |
| 1864 ............. | 1673 | 416 | 884 | 344 | 104 | 404 | 346 | 720 | 760 | 404 | 358 | 148 | 156 | 250 | 452 | 312 | 6058 |
| 1865............. | 1700 | 420 | 900 | 349 | 104 | 414 | 351 | 721 | 768 | 434 | 408 | 157 | 167 | 250 | 461 | 318 | 6217 |

This table gives the exact number of children that died in this city at the various ages indicated therein, also still-born in the several years. I have been unable to make comparisons of similar deaths in other cities, there being no classification (so minute) to my knowledge made. I hope the time is not far distant when a system of this character will be adopted in all cities and countries where mortuary statistics are or may be kept. In estimating the benefits of the progress of medical science and the results of sanitary improvements in reducing infantile mortality, the only correct basis is the *number* of deaths in a given period compared with another of a certain population. The causes of death are important; but the fact that diseases change at different periods, and physicians do not always call the same disease by the same name, and farther, it is not an easy matter in all cases to give the precise disease of which very young children die, therefore the cause of death cannot be so easily determined, but the age can almost always be ascertained.

